# The riddle of orange–red luminescence in Bismuth-doped silica glasses

**DOI:** 10.1038/s41598-021-87290-z

**Published:** 2021-04-08

**Authors:** Oleksii V. Laguta, Igor M. Razdobreev

**Affiliations:** 1 Central European Institute of Technology, CEITEC BUT, Purkyňova 656/123, 61200 Brno, Czech Republic; 2 UMR 8523 - PHLAM - Physique des Lasers Atomes et Molécules, CERLA, Univ. Lille, CNRS, 59000 Lille, France

**Keywords:** Lasers, LEDs and light sources, Optical materials and structures

## Abstract

For over the past two decades it has been believed that the intense orange-red photoluminescence in Bismuth-doped materials originates from Bi$^{2+}$ ions. Based on the results from magnetic circular polarization experiments, we demonstrate that this hypothesis fails for Bismuth-doped silica glasses. Our findings contradict the generally accepted statement that the orange-red luminescence arises from $^{2}P_{3/2}(1)$ $\rightarrow$ $^{2}P_{1/2}$ transition in a divalent Bismuth ion. The degree of magnetic circular polarization of this luminescence exhibits non-monotonic temperature and field dependencies, as well as sign reversal. This complex behaviour cannot be explained under the assumption of a single Bi$^{2+}$ ion. The detailed analysis enables us to construct a consistent diagram of energy levels involved in the magneto-optical experiments and propose a new interpretation of the nature of orange-red luminescence in Bismuth-doped silica glass. A centre responsible for this notorious photoluminescence must be an even-electron system with an integer total spin, presumably a dimer of Bismuth ions or a complex consisting of Bi$^{2+}$ and an oxygen vacancy.

## Introduction

The orange-red photoluminescence (ORPL) in Bismuth-doped materials has been noted since the work of Lecoq de Boisbaudran^1^, who observed this luminescence in MSO$_{4}$:Bi (M = Zn, Cd and Pb) sulfates which were subjected to the electrical discharge. Nevertheless, only at the end of the 20th century did Blasse et al.^2,3^ attribute the origin of this unusual ORPL in Bismuth-doped strontium tetraborate SrB$_{4}$O$_{7}$:Bi and some alkaline-earth-metal sulfates to a $^{2}P_{3/2}$ $\rightarrow$ $^{2}P_{1/2}$ transition in a divalent Bismuth ion. In the following years, a wide range of Bi-doped materials exhibiting the ORPL was synthesized – barium borates^4^, phosphates^5–7^, fluorides^8^, barite^9^, grossite^10^. The main reason for such increased interest in these materials is the potential use of the ORPL to improve the efficiency and colour temperature of white light-emitting diodes^11–14^. Notably, the authors of the above-mentioned works assigned the orange-red photoluminescence to a Bi$^{2+}$ ion based exclusively on the resemblance of the absorption/emission spectra to those observed by Blasse et al.^2^.

The electronic configuration of a Bi$^{2+}$ ion, $6s^{2}6p^{1}$, in principle, suggests that an EPR signal should be observed. However, even at liquid helium temperature, the EPR signal could not be observed in any of the studied compounds. De Jong et al.^15^ have recently re-examined the ORPL in SrB$_{4}$O$_{7}$:Bi in order to ascertain the reason why the EPR signal is not observed. According to the authors, the very low upper limit of the Bi$^{2+}$ concentration, which was estimated to be 20 ppm, is the main reason that the EPR signal in this material cannot be detected. It is worth noting, however, that in their analysis, it was a priori postulated that Bi$^{2+}$ is responsible for the ORPL.

In this regard, it should be noted that at least two Bi-doped tungstates, namely, CdWO$_{4}$^16^ and PbWO$_4$^17^, are known in which Bi$^{2+}$ related EPR was investigated. The EPR signal in these single crystals appears only after irradiation (X-rays, mercury or xenon lamps) at low temperatures (77 – 100 K) and it completely disappears at room temperature. Unfortunately, both compounds exhibit intrinsic ORPL, which is observed in these materials under excitation in the range of 340 – 370 nm (for PbWO$_{4}$ see, for instance ref.^18^), while the appearance of new additional photoluminescence (PL) or absorption bands directly connected to Bi$^{2+}$ ions was not reported and remains questionable.

Another class of materials that exhibit ORPL related to Bismuth doping are silica-based glasses. The interest in these materials arose due to the discovery of Bismuth-related broadband near infrared photoluminescence (NIR PL)^19,20^. Since silica-based glasses are compatible with modern fibre optic systems, tremendous progress has been made over the past 20 years in the development of bismuth-based fibre lasers and amplifiers^21–31^. Nevertheless, despite this technological progress, the nature of NIR PL and lasing centres continues to be discussed^32–34^.

The intense ORPL observed under UV or blue-green excitation is also one of the intriguing features of Bismuth-doped fibres. This PL was demonstrated for the first time in bulk sintered fibre preforms fabricated from nano-porous xerogels^35^. Later, this luminescence was attributed to a single Bi$^{2+}$ ion by analogy with the work of Blasse et al.^2^ and at present, to the best of our knowledge, this is a generally accepted interpretation^28,36–38^.

The ORPL in Bi-doped silica glasses is observed after high-temperature sintering of the porous host matrix or after the melting of raw compounds, and its recording does not require any irradiation. At the same time, all our attempts to detect the EPR signal after irradiation at low temperatures (down to 5 K) had failed. For this reason, the identification of ORPL in Bismuth-doped silica glass as a transition $^{2}P_{3/2}$ $\rightarrow$ $^{2}P_{1/2}$ in a divalent Bismuth ion was doubtful.

In our recent paper^39^, we investigated the magnetic circular dichroism (MCD) in bulk fibre preforms SiO$_2$:Bi, in which both centres, ORPL and NIR PL (lasing centre), coexist. It was shown that the recorded MCD bands, most probably, should be assigned to the ORPL related centre. Furthermore, the MCD behaviour as a function of magnetic field and temperature can be explained only by assuming the even-electron nature of a single emitting centre. Thus, the latter cannot be identified as a Bi$^{2+}$ ion. Unfortunately, even at very low doping levels Bismuth-doped glasses are always characterized by the multiplicity of optical centres and the unambiguous correspondence between the absorption and emission bands is not obvious. For this reason, in the present paper, we report on the measurements of magnetic circular polarization of luminescence (MCPL) in bulk Bismuth-doped silica glass. MCPL directly characterizes the magnetic properties of the excited states and offers such advantages over the standard EPR as high sensitivity and selectivity. In contrast to our earlier study^40^, in which we investigated the NIR PL of the lasing centre, here we analyse the ORPL and accompanying PL in the region of 850 nm. The results of the present work constitute direct and unequivocal evidence that the ORPL in Bi-doped silica glass cannot be identified as a $^{2}P_{3/2}$ $\rightarrow$ $^{2}P_{1/2}$ transition in a Bi$^{2+}$ ion. Also, based on the results obtained from MCPL and MCD experiments, we have constructed a consistent energy level diagram of the ORPL centre and discuss its nature.

## Results

### Photoluminescence and excitation spectra

**Figure 1:**
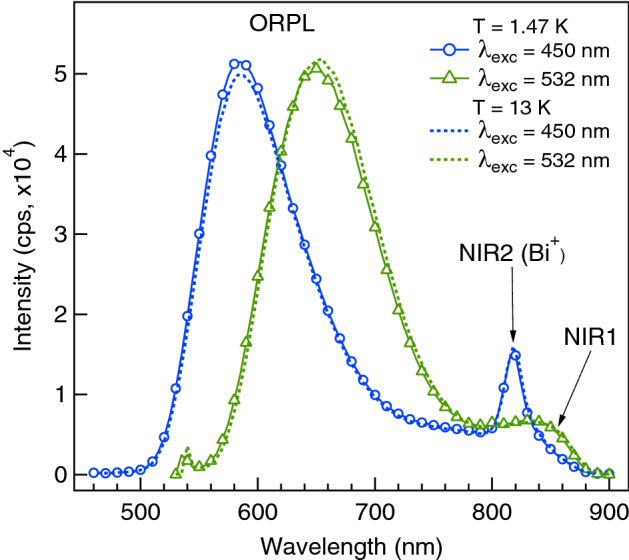
Photoluminescence spectra of SiO$_2$:Bi at different excitation wavelengths and temperatures in the magnetic field 6 T.

In Fig. 1 we show the PL spectra recorded in the range of 460 – 900 nm under excitation at 450 and 532 nm. The spectra were recorded at two temperatures: 1.47 and 13 K. Although all the spectra shown in Fig. 1 were recorded in a magnetic field of 6T, we did not reveal any field dependence of the PL spectrum. It is seen that the PL spectrum at both excitation WLs consists of the intense ORPL band accompanied by a long-wavelength tail. The strong excitation wavelength dependence of the ORPL is due to its non-homogeneous broadening. The maximum intensity of the ORPL band is at 585 and 650 nm, while its full width at half maximum is approximately 3000 and 2600 cm$^{-1}$ under excitation at 450 and 532 nm, respectively. The change of temperature only slightly affects the shape and position of the ORPL, being within the experimental accuracy. It is very important to note that under excitation at 532 nm a relatively weak and broad PL band NIR1 with the maximum around 850 nm accompanies the ORPL. As it will be shown below, this NIR1 and the ORPL bands belong to the same luminescent centre. Like the ORPL band, the NIR1 band also exhibits strong non-homogeneous broadening. The additional relatively narrow band NIR2 at 830 nm, which is observed under excitation at 450 nm, belongs to the lasing centre and should be assigned to the $2\Pi \longrightarrow 1\Sigma ^+$ transition in a Bi$^+$ ion, which has been discussed by us previously^34^.

**Figure 2:**
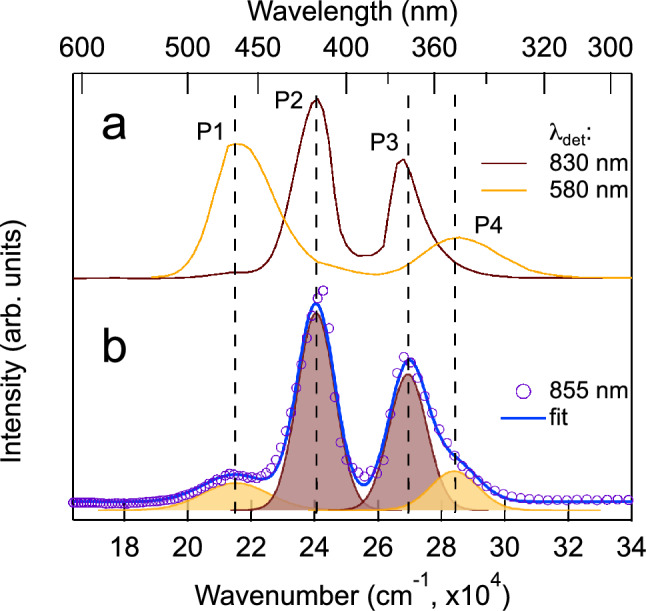
Excitation spectra. (**a**) PL excitation spectra of the ORPL and NIR2 bands. (**b**) PL excitation spectrum of the NIR1 band recorded at 855 nm and its multi-peak deconvolution. All the spectra were recorded at 1.47 K and zero magnetic field.

Such an assignment is also supported by the measurements of excitation spectra shown in Fig. 2. It is seen in Fig. 2a that the excitation spectra recorded at 580 and 830 nm are very different. The excitation spectrum of the ORPL band recorded at 580 nm shows two well-separated peaks (P1 and P4). The spectrum recorded at 830 nm consists of two strong peaks (labelled P2 and P3) which are caused by the transitions $$1\Sigma ^+ \longrightarrow 3\Sigma ^+$$ (P2) and $$1\Sigma ^+ \longrightarrow 3\Pi$$ (P3) in Bi$$^+$$ ^34^. The short- and long-wavelength tails, though, seem to coincide with P1 and P4 peaks of the ORPL spectrum, and are due to the overlapping of NIR2 and NIR1 luminescence. Finally, the excitation spectrum recorded at 855 nm shown in Fig. 2b can be considered as a weighted sum of the two previous spectra. Its shape is again due to the overlapping of the luminescence spectra of two different centres: the Bi$$^+$$ ion and the centre under the question responsible for the ORPL. However, at 855 nm the relative PL intensities of these centres are comparable because in Bi$$^+$$ at this particular wavelength the active transition is $$2\Sigma ^+ \longrightarrow 1\Sigma ^+$$, which is much weaker than the $$2\Pi \longrightarrow 1\Sigma ^+$$ transition at 830 nm (see Fig. 1 and Table 1 in Ref.^34^). The presence of the additional spectral band NIR1, which belongs to the ORPL centre, becomes even more obvious when studying the MCPL.

### Magnetic circular polarization of luminescence

**Figure 3:**
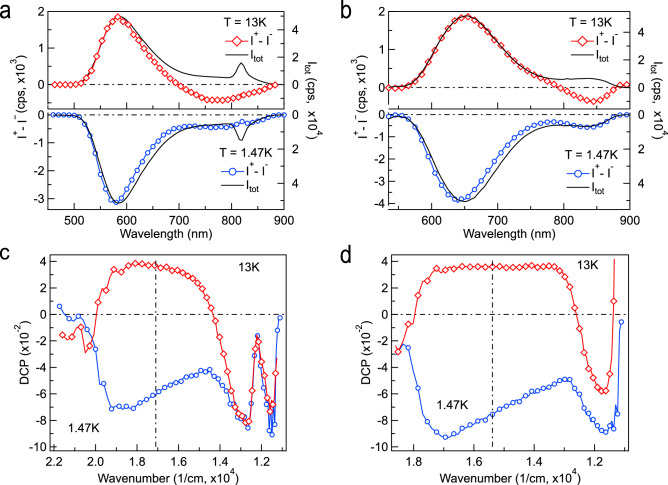
Comparison of MCPL ($I^{+} - I^{-}$) and total intensity spectra recorded upon excitation at 450 and 532 nm ( (**a**) vs (**b**) and (**c**) vs (**d**)). Comparison of DCP $(I^{+} - I^{-})/(I^{+} + I^{-})$ spectra recorded at temperatures of 1.47 and 13 K. Magnetic field is 6 T in all panels. Vertical dash-dot lines indicate the band maximum position.

In Fig. 3a,b we show the MCPL spectra ($I^{+}- I^{-}$) along with spectra of the total intensity ($(I^{+}+ I^{-})/2$) recorded in a magnetic field of 6 T at two temperatures: 1.47 and 13 K. The most noticeable feature is the different sign of circular polarization in the ORPL band at these temperatures; namely, it is negative at the lower temperature and positive at the higher. Additionally, at the lower temperature the peak of MCPL is blue-shifted relative to the PL band for both excitation WLs. This shift becomes imperceptible at T = 13 K. It is also seen that the MCPL in the long-wavelength tail of ORPL is negative at both temperatures and it does not correlate with the narrow PL band observed at 830 nm. It follows that this broad PL should be attributed to a separate band, which is not related to the luminescence of Bi$^{+}$ ions, and, on the contrary, belongs to the ORPL centre.

In the study of MCPL, the most useful quantity is the so-called emission anisotropy factor or the degree of circular polarization (DCP), which is defined as $DCP = (I^{+} - I^{-})/(I^{+} + I^{-})$^41,42^. Similarly to MCD, this quantity can be presented as a sum of three terms: $\mathscr {A}$, $\mathscr {B}$ and $\mathscr {C}$. For the simplest model of an isolated Gaussian band, only the $\mathscr {A}$-term exhibits a spectral dependence: $\mathscr {A} \sim f(\nu - \nu _{0})$. Then, the observed DCP spectrum, as a rule, takes the form of an inclined ($\mathscr {A} \ne 0$) or a horizontal ($\mathscr {A} = 0$) straight line^41^. A very useful property of the $\mathscr {A}$-term is that it takes zero value at the maximum intensity of the PL band^43^. In the vast majority of cases, $\mathscr {A}$- and $\mathscr {B}$-terms are temperature independent; therefore, measurements of DCP at the maximum PL band intensity allow the direct study of the paramagnetic term $\mathscr {C}$. The experimental spectra of DCP recorded in the magnetic field of 6 T at the excitation WLs 450 and 532 nm, are shown in Fig. 3c,d, respectively. It is seen that at the low temperature the DCP spectrum of the main ORPL band can be well approximated by a single inclined straight line. The parameters that correspond to the paramagnetic $\mathscr {C}$ and diamagnetic $\mathscr {A}$ terms obtained from the fit are as follows: a) $\mathscr {C} = - 5.99(1) \times 10^{-2}$ and $\mathscr {A} = - 8.74(1) \times 10^{-6}$, and b) $\mathscr {C} = - 7.52(2) \times 10^{-2}$ and $\mathscr {A} = - 1.14(1) \times 10^{-5}$ for the above excitation WLs, respectively. At a temperature of 13 K, the paramagnetic terms become positive and are very close for both excitation WLs: $\mathscr {C} = 3.5(1) \times 10^{-2}$, while the $\mathscr {A}$-term becomes negligible. This strange behaviour of the $\mathscr {A}$-term (by the definition it is temperature insensitive) can appear only if the MCPL originates from two or more magnetic multiplets, and it is well explained in our model presented in the next section. There is another important feature of the observed DCP that must be pointed out. It is seen that the long-wavelength tail of luminescence always exhibits negative DCP. The sharp peak, which can be seen in Fig. 3c at 12050 cm$^{-1}$ (830 nm), can be also observed under excitation at 375 nm, where PL, and consequently positive DCP, from Bi$^{+}$ ions become dominant^40^. This peak is absent in the DCP spectrum recorded at $\lambda _{exc}$ = 532 nm (panel (d) of Fig. 3), while the negative DCP in this spectral region practically reproduces that in panel (c) of Fig. 3. Thus, the measurements of MCPL confirm that the PL NIR2 is due to Bi$^{+}$ ions and it appears due to the partial overlap of its excitation spectrum and that of ORPL, as it was pointed out previously in this work.

**Figure 4:**
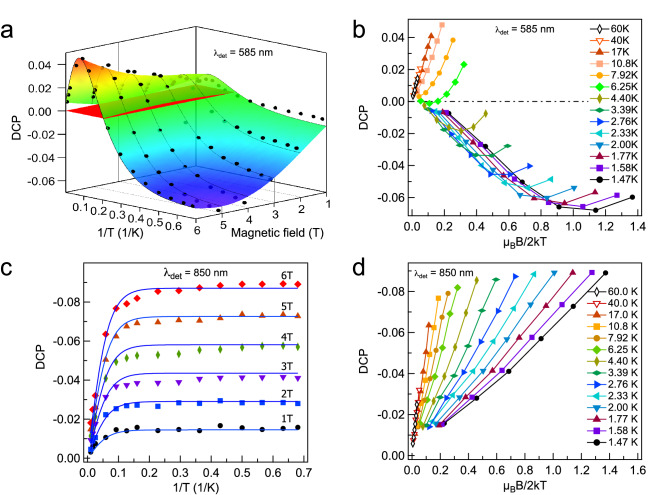
DCP data recorded at 585 and 850 nm upon excitation at 450 and 532 nm, respectively. (**a**) Data are shown as a function of temperature at fixed magnetic fields. Black dot markers— experimental data, rainbow-coloured surface—theoretical DCP calculated using parameters from the data fit to Eq. (1) with $i=2$. The red colour plane indicates the zero polarization level. b) Magnetic field dependence of DCP at fixed temperatures. (**c**) Data recorded at 850 nm are shown as a function of temperature at fixed magnetic fields. Markers—experimental data, lines— theoretical DCP calculated from the data fit to Eq. (1) with $i=1$. (**d**) DCP data shown as a function of magnetic field at fixed temperatures.

Further detailed investigations of the MCPL in the ORPL band at a variable temperature and magnetic field (VTVH) were performed at 650 and 585 nm, corresponding to the maximums of ORPL at the lowest temperature under excitation at 532 and 450 nm, respectively. The experimental data on VTVH-MCPL obtained at 585 nm VTVH-MCPL are shown in Fig. 4a,b. In the left (a) panel the data are presented so as to show the temperature dependence at a fixed magnetic field. On the contrary, in the right panel Fig. 4b, the data is shown as a function of the magnetic field at a fixed temperature.

It is seen that at any fixed value of the magnetic field, DCP behaviour is non-monotonic. The degree of circular polarization increases with the temperature decrease and reaches its maximum value $\hbox {DCP} = + 0.047$ at T $\approx$ 10 K in the magnetic field of 6 T. Then, the DCP decreases, changes its sign in the vicinity of $\hbox {T} = 6$ K, and saturates. Another interesting detail is that the MCPL saturates to different values for each magnetic field magnitude. This is a distinguishing feature of an even-electron system^40,44^. Saturation curves recorded at 650 nm under excitation at 532 nm exhibit a rather similar temperature behaviour, and can be found in Fig. S1 of the Supplementary Materials.

We also performed VTVH measurements of DCP at the wavelength of 850 nm upon excitation at 532 nm to make only the ORPL centre active. As it is shown in Fig. 4c, the DCP exhibits a simple monotonic temperature dependence at any magnetic field. The polarization degree increases with temperature and saturates below 5 K. Similarly to the ORPL band, the value of the saturation parameter increases with the increasing magnetic field. In Fig. 4d we show the magnetic field dependences at fixed temperatures. It is seen that the magnetization curves exhibit the nested (or fan-out) behaviour characteristic of a zero-field split paramagnetic initial state. Thus, both representations of DCP behaviour shown in Fig. 4c,d directly indicate the even-electron system nature of the ORPL centre, whereas Bi$^{2+}$ has only one unpaired electron.

When analysing MCPL measured in the experiments with continuous wave (CW) excitation, the spin relaxation process must be taken into account. It is crucial that the population distribution reaches its thermodynamic equilibrium before the electrons relax from the excited state. This means that the spin-lattice relaxation time must be much shorter than the radiative decay time. If this condition is not satisfied, the obtained values of g-factor and ZFS will be incorrect. To ensure that in our experiments the above-mentioned condition is satisfied, we performed the measurements of time-resolved DCP^45^. In this technique, one records separately the time-resolved decays of $\sigma ^+$ and $\sigma ^-$ components of photoluminescence. Then the subsequent subtraction and normalization allow the obtainment of the time evolution of DCP. However, experiments with nanosecond time resolution did not allow us to observe the initial build-up of DCP after pulsed excitation, as it is shown in Fig. S2. In all our experiments DCP reached its equilibrium value instantly. This indicates a very fast spin relaxation, most probably at the picosecond scale, so that it does not influence the results of CW DCP measurements.

### MCPL analysis and energy level diagram

The unusual behaviour of DCP described above could be explained with the assumption that there are two independent even-electron centres, each of which contributes to luminescence. However, our earlier results on magnetic circular dichroism contradict this assumption^39^. Another possibility is to assume that there is only one kind of luminescent centre, in which case the ORPL is caused by the simultaneous transitions from at least two closely spaced excited states (or sub-states). The MCPL due to these excited states is opposite in sign and, in addition, the absolute values of their zero-field splitting (ZFS) must be significantly different.

**Figure 5:**
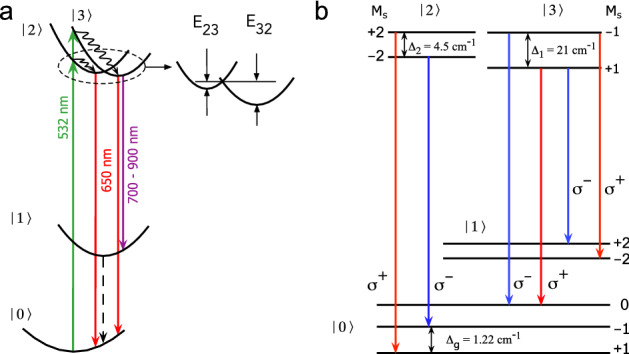
Energy levels diagram of the ORPL centre in SiO$_2$:Bi. (**a**) General layout. Wavy arrows correspond to fast non-radiative relaxation. Dashed arrow: possible, but unobserved yet transition. (**b**) Magnetic sublevel structure. All sublevels are labelled by the quantum numbers $M_S$ corresponding to the quantized projections of the spin *S*.

In Fig. 5 we show the energy level diagram of the ORPL centre, which satisfies all the observed MCPL features. Figure 5a gives a general outline of levels. Due to the strong inhomogeneous broadening, both states ${\vert }{2}{\rangle }$ or ${\vert }{3}{\rangle }$ can be excited simultaneously, but in the centres from different sub-sets. After the excitation, which is followed by fast non-radiative relaxation, the ORPL arises as a transition from these states to the ground state ${\vert }{0}{\rangle }$, while the PL band around 850 nm arises due to the transition from the state ${\vert }{3}{\rangle }$ to an intermediate state ${\vert }{1}{\rangle }$. Obviously, if the energy barriers E$_{23}$ and E$_{32}$ between the states ${\vert }{2}{\rangle }$ and ${\vert }{3}{\rangle }$ are sufficiently high (E$_{23}$, E$_{32}$ $\gg 1/kT$), then the DCP measured in the ORPL band can be represented as a sum of individual contributions.

Assuming that the MCPL is caused only by *xy*-polarized electric dipole transitions, and $g_{\parallel }\gg g_{\bot }$, the paramagnetic $\mathscr {C}$-term of DCP is^40^:

1 $$\begin{aligned} \mathscr {C}(T,B) = \sum _{i=1}^{N}A_i^{sat}\int _0^1\!\frac{\tilde{g}_i\mu _BBn^4}{\sqrt{\Delta ^2_i+(\tilde{g}_i\mu _BBn)^2}}\tanh \left( \frac{\sqrt{\Delta ^2_i+(\tilde{g}_i\mu _BBn)^2}}{2kT}\right) \mathrm {d}n, \end{aligned}$$

where the summation is over all the transitions from different excited states contributing at the given WL, $n = \cos (\theta )$, A$_{i}^{sat}$ are the saturation constants, $\Delta _{i}$ are the corresponding ZFS’s between two magnetic sub-levels due to the low symmetry component of the crystal field, $\tilde{g}_{i}$ are the effective g-factors, which, for example, are equal to $4g_{\parallel ,i}$ or $2g_{\parallel ,i}$ for a spin quintet ($S = 2$) and a spin triplet ($S = 1$), respectively. This expression is valid for any set of strongly anisotropic and non-interacting (term $\mathscr {B} = 0$) doublets, Kramers or non-Kramers. If some particular transition involves half-integer spin states, then the corresponding ZFS energy $\Delta _{i}$ rigorously equals zero.

**Table 1:** Zero field splittings (ZFS), saturation constants and effective $\tilde{g}$-factors for ORPL and NIR PL (Bi$^+$ ion) centres in SiO$_2$:Bi glass. The excitation WL for the MCPL is given in parentheses.

	Bi$^+$	ORPL centre	$A^{sat}$	$\tilde{g}$	ZFS ($\Delta$)
	Wavelength, nm	Wavelength, nm			cm$^{-1}$
MCD	–	465	$(2.8 \pm 0.03)\times 10^{-3}$	$2.16 \pm 0.04$	$1.22 \pm 0.11$
MCPL		580 (450)	$-1.8 \pm 0.3$	$1.4 \pm 0.16$	$5.1 \pm 0.4$
			$11 \pm 0.6$	$0.44 \pm 6$	$17 \pm 2$
		640 (532)	$-1.4 \pm 0.2$	$1.28 \pm 0.14$	$4.5 \pm 0.3$
			$7.6 \pm 0.5$	$0.56 \pm 8$	$21 \pm 2.5$
		850 (532)	$-7.6 \pm 0.8$	$0.86 \pm 2$	$21 \pm 3$
	830 (375)		$1.26 \pm 0.08$	$1.4 \pm 10$	$97 \pm 6$
	1440 (375)		$-1.38 \pm 0.06$	$4.28 \pm 0.17$	$6.18 \pm 0.24$

The two-dimensional fit of the experimental data set DCP(T,B) to the Eq. (1) is shown in Fig. 4a for WL 585 nm. It can be seen that our simplified model simulates the temperature and magnetic field dependences reasonably well, though, it can be further improved by taking into account possible transitions between states ${\vert }{2}{\rangle }$ and ${\vert }{3}{\rangle }$. The parameters of spin Hamiltonian for the corresponding excited states are given in Table 1, which, for the sake of completeness, is supplemented by the results obtained from the MCD measurements (ORPL centre) and MCPL in the lasing centre (presumably, Bi$^{+}$ ion)^34,39,40^. It is worth noting, that with the ZFS parameter fixed to zero, it was impossible to get any satisfactory fit. This allows us to make the unequivocal conclusion that a single Bi$^{2+}$ ion or, as a matter of fact, any other system with a half-integer spin cannot cause this orange–red luminescence.

The right panel in Fig. 5 shows the fine structure of energy levels. This model depicts the simplest case with the triplet ground and second excited states since neither MCD nor MCPL gives the exact value of the effective spin *S*. One can see that the negative MCPL component originates from the excited state with the ZFS $\Delta =4.5$ cm$^{-1}$, while the positive one from the state with $\Delta =21$ cm$^{-1}$. The effective $\tilde{g}$-factor of the excited state with a high ZFS (see Table 1) is small and it has a large fit uncertainty. This is not surprising, since the main contribution to the splitting of doublet sub-levels is due to ZFS, while the Zeeman term in the available range of magnetic fields is not sufficiently large for the precise g-factor determination. It is also shown in Table 1 that the set of parameters obtained from the DCP data for NIR1 band (850 nm) is practically identical to that of the positive DCP component of ORPL. This is very strong evidence that these transitions have the same initial excited sub-state and different terminating states resulting in opposite DCP signs. According to the model of Fig. 5b, the NIR1 band is caused only by the transition from the state ${\vert }{3}{\rangle }$ to the first (intermediate) excited state ${\vert }{1}{\rangle }$. The subsequent transition from ${\vert }{1}{\rangle }$ to the ground state, if it exists, should produce NIR luminescence beyond 2 $\upmu$m, and perhaps this is the reason why it has not been reported so far.

The proposed energy levels diagram nicely explains the observed zeroing of the $\mathscr {A}$-term at 13 K in the ORPL band. We recall that the A-term is temperature independent according to the standard theory^41–44^. For this reason, we believe that the observed $\mathscr {A}$-term should be considered as a *pseudo* $\mathscr {A}$-term, the appearance of which is associated with two overlapping $\mathscr {C}$-terms of opposite signs^46^. First, according to Fig. 5b, only the transition ${\vert }{2}{\rangle } \longrightarrow {\vert }{0}{\rangle }$ produces a genuine $\mathscr {A}$-term as a result of the ground state splitting. However, due to the relatively small value of this splitting, $\Delta W$, on the one hand, and the large width of the luminescence band, $\Delta \nu$, on the other hand, we would expect the diamagnetic term to be negligibly small even at high magnetic fields since $\mathscr {A} \simeq \Delta W/\Delta \nu$. Second, in our model, the states ${\vert }{2}{\rangle }$ and ${\vert }{3}{\rangle }$ have the same excitation probabilities, meaning that they are equally populated at low temperatures, and the transitions to the ground state are of comparable intensities. However, the absolute degree of polarization is higher for emission from the state with a small ZFS, i. e. state ${\vert }{2}{\rangle }$. This is because the DCP is inversely proportional to the zero-field splitting $\Delta$ at low and intermediate magnetic fields ($\tilde{g}\mu _BB \le \Delta$). As the result, the DCP is negative and has a non-zero slope at 1.47 K. As the temperature rises, the $\mathscr {C}$-term of state ${\vert }{2}{\rangle }$ decreases faster than that of state ${\vert }{3}{\rangle }$, reducing the pseudo $\mathscr {A}$-term. Additionally, the possible relaxation ${\vert }{2}{\rangle } \longrightarrow {\vert }{3}{\rangle }$ over the barrier E$_{23}$ contributes to the redistribution of the excited states’ populations in favour of the transition ${\vert }{3}{\rangle } \longrightarrow {\vert }{0}{\rangle }$. This process can also decrease the contribution of the state ${\vert }{2}{\rangle }$ to the DCP, which leads to zeroing of the pseudo $\mathscr {A}$-term.

According to the selection rules, the sign of MCPL is defined by the change of the magnetic moment’s projection $\mu _z=-\mu _BgM_S$ during the transition between the energy levels. Since in our model the final state is the same and has the zero projection ($M_S=0$), the two components of MCPL can have the opposite signs only if the corresponding excited states have the opposite moments $\mu _z$. Consequently, the negative MCPL, e.g. the left-hand polarized component $\sigma ^-$, originates from the state with a positive $\mu _z$, and the $\sigma ^+$ component from the state with a negative $\mu _z$. In terms of the spin Hamiltonian it means that the non-Kramers doublet ${\vert }{\pm 1}{\rangle }$ of the state ${\vert }{3}{\rangle }$ has a negative g-factor.

The appearance of a negative g-factor is possible in a system with large spin *S* and interactions that introduce non-diagonal terms to the spin Hamiltonian. A simple example is a Yb$^{3+}$ ion in the octahedral environment^47^. Chibotaru and Ungur^48^ discussed possible mechanisms for negative g-factors in lanthanide and transition metal complexes. They concluded that the necessary condition for a negative g-factor is the presence of strong fourth- and higher-order perturbations, namely the ZFS effects and/or strong spin-orbit coupling. Returning to Bi$^{2+}$, it indeed exhibits a very strong spin-orbit interaction, which splits the $^2$P term of the 6*p* electron to the ground state $^2$P$_{1/2}$ and the first excited state $^2$P$_{3/2}$. However, the fourth- and higher-order perturbation effects do not arise for the states of quartet and lower multiplicity^47,49^. This is yet another fact excluding Bi$^{2+}$ as the origin of the ORPL in Bismuth-doped silica glass.

Another class of systems that can demonstrate a negative g-factor is clusters of ions. The exchange coupling mixes the states of individual ions producing similar effects as high-order spin-orbit and crystal field interactions. Piligkos et al.^50^ investigated transition metal dimers with a weak exchange coupling using MCD spectroscopy. Among different model systems they observed a reverse of the MCD signal sign in an antiferromagnetically coupled Cr(III)Ni(II) dimer at the temperatures above $\simeq$15 K. The ground state of the system consists of three sub-levels with the lowest spin state S $=$ 1/2. The other two spin states of the system, S $=$ 3/2 and S $=$ 5/2, lie at 20.1 and 53.6 cm$^{-1}$ above the lowest one, respectively. Magnetic moments of the excited states are opposite to the magnetic moment of the S $=$ 1/2 ground doublet. Above 15 K the population of excited states becomes sufficiently high to produce a major contribution to MCD, which reverses its sign.

Taking into account the above considerations, it should be clear that the orange–red luminescence can not be assigned to a single Bi$^{2+}$ ion. This active centre must have a more complex electronic structure. The simplest objects that could satisfy the features observed in the MCD and MCPL experiments are, for example, homo-nuclear dimers. It is worth recalling here that Bismuth ions are characterized by extended outer *p*-orbitals, which enhances a tendency for clustering. On this basis, it can be assumed that divalent Bismuth ions compose a dimer [Bi$^{2+}$— Bi$^{2+}$] that is responsible for the orange–red photoluminescence. Alternatively, one can assume that some kind of a stable complex formed by a Bi$^{2+}$ ion and an oxygen vacancy ([Bi – V$_{O}$] $^{n+}$) is the origin of the ORPL. Unfortunately, MCPL is not capable of distinguishing these two cases. As a solution, the optically detected nuclear magnetic resonance can be used. This technique can provide the same information as the conventional NMR but it is more selectable since it probes the nuclei of the luminescence centre only^51,52^. On the other hand, ORPL is observed even at very low doping levels of Bismuth ($\sim$1 ppm) at which forming of dimers is quite improbable, and therefore the second assumption seems to be more realistic.

## Discussion and conclusions

Detailed investigations of magnetic circular polarization of the orange–red photoluminescence band in Bismuth-doped silica glass without other co-dopants were performed at variable temperature and magnetic field. The experiments show rather complex behaviour of the degree of circular polarization, which exhibits a non-monotonic temperature and field dependence, and sign reversal. Such behaviour cannot be explained by the generally accepted assumption of Bi$^{2+}$ being the origin of the ORPL. The analysis of VTVH curves revealed that the excited states of the ORPL centre are magnetic multiplets with large zero-field splitting. We proposed a consistent model of energy levels of the ORPL centre, which explains all the peculiarities observed in MCPL and MCD experiments. We assume that the source of this thermally stable orange–red photoluminescence can be homonuclear Bismuth dimers ([Bi$^{2+}$ – Bi$^{2+}$], for instance) or complexes consisting of a Bi$^{2+}$ ion and an oxygen vacancy [Bi – V$_{O}$] $^{n+}$, and each of these centres must be a system with an even number of electrons.

## Methods

### Materials

In the present investigation, two types of material were used. The first type of the samples was manufactured using the melting of SiO$_{2}$ and Bi$_{2}$O$_{3}$ powders in silica tubes^53^. The size of particles was 100–200 and 1–5 $\upmu$m for silica and bismuth oxide, respectively. The mass ratio of the oxides was chosen so that the atomic ratio Bi/Si obtained in the glass was about of 5000 ppm. The detailed procedure of sample fabrication was very close to that described previously by I. Bufetov et al.^54^.

The second type of material used in our experiment was a Bismuth-doped silica glass preform manufactured from nano-porous silica xerogels. The xerogels were doped with bismuth by soaking in an acetone solution of Bi-containing precursor complex. After the dehydroxylation, the xerogels were sintered at 1300 $^{\circ }$C in a helium atmosphere. A more detailed description of this material fabrication was reported elsewhere^35,55,56^. The samples prepared from this material were the same that we previously used in the MCD experiments^39^, and we recall here that the atomic ratio Bi/Si estimated from the standard electron probe microanalysis (EPMA) was about 400 ppm.

Both types of samples were cut and polished to get the dimensions of about $2\times 4\times 5$ mm$^{3}$. The samples showed very similar results. We presented here the data obtained from the second type of material only since it was used in the MCD measurements reported before^39^.

### Measurements

Experiments in the temperature range 1.4–300 K were performed in the closed cycle magneto-optical cryostat (SpectromagPT, Oxford Instr.). The thermal stability of the samples attached to the holder of the variable temperature insert was about 0.01 K, except the range from 4.2 to 10 K, where the thermal stability was about 0.05 K.

Laser diodes and frequency doubled Nd:YAG lasers were used for the excitation at different wavelengths. In the experiments with continuous wave excitation the quarter-wave retardation ($\lambda /4$) was introduced by the photoelastic modulator (I/FS-20, Hinds Instruments) at the frequency 20.077 kHz. The resulting PL emission in the range of 460–900 nm was analysed by the fixed uncoated Glan-Thompson polarizer, then filtered by a monochromator and detected with the cooled GaAs photomultiplier (R943-02, Hamamatsu Inc.). The photon counting technique (P7887 scaler, Fast ComTec) was used to record the signal because of its outstanding signal to noise ratio. In the experiments with pulsed excitation the photoelastic modulator was replaced with a super-achromatic $\lambda /4$ waveplate. Other equipment and details of the experiments were described elsewhere^40^. The spectral resolution in the investigated WL range was 1.5 nm.

## Supplementary information

Supplementary Information

## Data Availability

Experimental data shown in the graphs throughout this article are available from the corresponding author upon reasonable request.
