# Supplementary material for: The riddle of orange–red luminescence in Bismuth-doped silica glasses
Source: Sci Rep. 2021 Apr 8;11:7766. doi: 10.1038/s41598-021-87290-z (PMC8032712; doi:10.1038/s41598-021-87290-z)
Supplement: Supplementary file 1 — Supplementary Information [file 41598_2021_87290_MOESM1_ESM.pdf]

Supplementary Materials for "The riddle of orange-red luminescence in Bismuth-doped silica glasses"

Oleksii V. Laguta[1,*] and Igor M. Razdobreev[2,**]

[1] *Central European Institute of Technology, CEITEC BUT, Purkyňova 656/123, 61200 Brno, Czech Republic*

[2] *Univ. Lille, CNRS, UMR 8523 - PHLAM - Physique des Lasers Atomes et Molécules, F-59000, Lille, France*

[*] *oleksii.laguta@ceitec.vutbr.cz*

[**] *igor.razdobreev@univ-lille.fr*

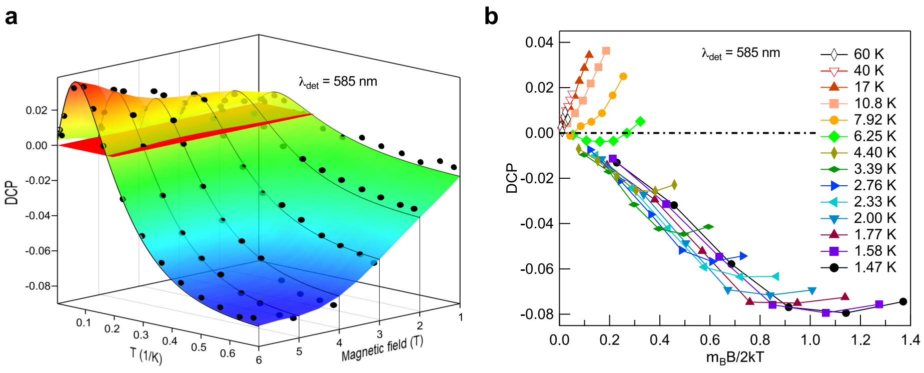

Figure S1: DCP data recorded at 640 nm upon excitation at 532 nm. a) Data are shown as a function of temperature at a fixed magnetic field. Black dot markers – experimental data, rainbow-coloured surface – theoretical DCP calculated using parameters from the data fit to Eq. 1 of the main text with $i = 2$. Red colour plane indicates the zero polarisation level. b) Magnetic field dependence of DCP at fixed temperatures.

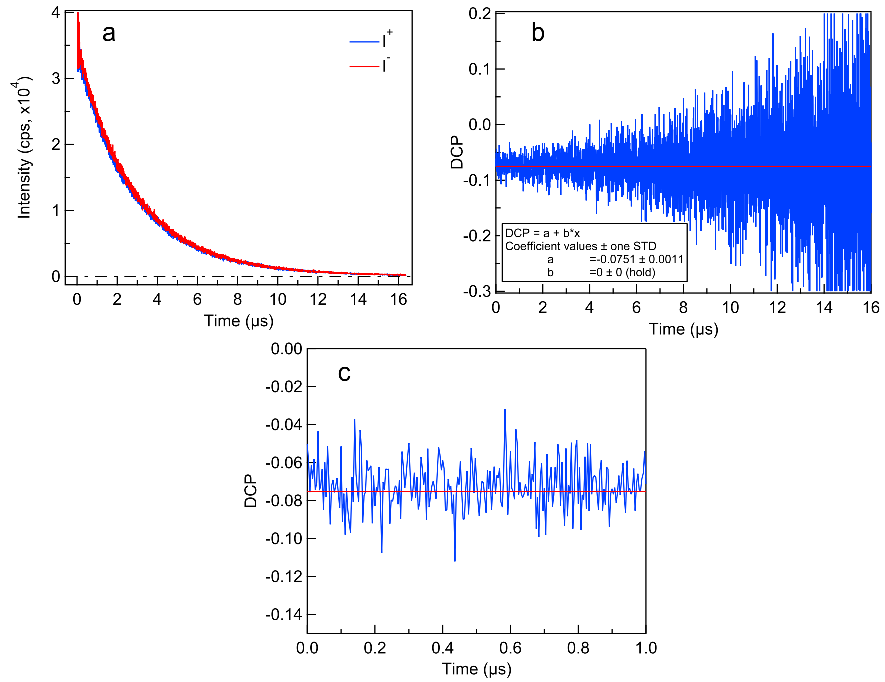

Figure S2: Time evolution of DCP recorded with 4 ns resolution at 640 nm upon excitation at 532 nm. a) Emission decay of σ^+ and σ^- components of the ORPL band. b) The DCP kinetics and its linear fit revealing the same polarisation value as in CW experiments. c) A magnified view of b) showing that the DCP reaches its equilibrium value virtually instantly after the laser pulse excitation.
